# Deep Learning-Enhanced Spectroscopic Technologies for Food Quality Assessment: Convergence and Emerging Frontiers

**DOI:** 10.3390/foods14132350

**Published:** 2025-07-02

**Authors:** Zhichen Lun, Xiaohong Wu, Jiajun Dong, Bin Wu

**Affiliations:** 1 School of Electrical and Information Engineering, Jiangsu University, Zhenjiang 212013, China; 2222407011@stmail.ujs.edu.cn (Z.L.); 2222407079@stmail.ujs.edu.cn (J.D.); 2 High-Tech Key Laboratory of Agricultural Equipment and Intelligence of Jiangsu Province, Jiangsu University, Zhenjiang 212013, China; 3 Department of Information Engineering, Chuzhou Polytechnic, Chuzhou 239000, China; 4 School of Computer Science and Engineering, Southeast University, Nanjing 211189, China

**Keywords:** quality inspection, spectroscopic technologies, deep learning, spectral-heterogeneous fusion, multimodal integration

## Abstract

Nowadays, the development of the food industry and economic recovery have driven escalating consumer demands for high-quality, nutritious, and safe food products, and spectroscopic technologies are increasingly prominent as essential tools for food quality inspection. Concurrently, the rapid rise of artificial intelligence (AI) has created new opportunities for food quality detection. As a critical branch of AI, deep learning synergizes with spectroscopic technologies to enhance spectral data processing accuracy, enable real-time decision making, and address challenges from complex matrices and spectral noise. This review summarizes six cutting-edge nondestructive spectroscopic and imaging technologies, near-infrared/mid-infrared spectroscopy, Raman spectroscopy, fluorescence spectroscopy, hyperspectral imaging (spanning the UV, visible, and NIR regions, to simultaneously capture both spatial distribution and spectral signatures of sample constituents), terahertz spectroscopy, and nuclear magnetic resonance (NMR), along with their transformative applications. We systematically elucidate the fundamental principles and distinctive merits of each technological approach, with a particular focus on their deep learning-based integration with spectral fusion techniques and hybrid spectral-heterogeneous fusion methodologies. Our analysis reveals that the synergy between spectroscopic technologies and deep learning demonstrates unparalleled superiority in speed, precision, and non-invasiveness. Future research should prioritize three directions: multimodal integration of spectroscopic technologies, edge computing in portable devices, and AI-driven applications, ultimately establishing a high-precision and sustainable food quality inspection system spanning from production to consumption.

## 1. Introduction

Food safety and quality have become increasingly pivotal in modern society due to their direct implications for consumer health and global sustainable development [1]. Studies project a 35–56% growth in global food demand between 2010 and 2050, alongside substantial fluctuations in undernourished populations (−91% to +8%) under varying socioeconomic and climate scenarios [2]. Nevertheless, the impact of foodborne diseases (FBDs) in low-income and middle-income countries (LMICs) is comparable to malaria, with over 90% of the global FBDs load falling on these regions. The economic cost exceeds USD 100 million annually [3]. Food fraud, characterized by sophisticated practices such as species substitution and misdeclaration of geographical origin, continues to threaten consumer welfare and market trust. While 70% of nations have implemented preventive measures, merely 13% have completed vulnerability assessments, and over half delegate oversight of this issue to non-food safety agencies, exposing systemic regulatory deficiencies [4,5,6]. Concurrently, agricultural intensification, environmental stressors, and limited crop knowledge are driving nutritional quality decline in crops, particularly micronutrient depletion in fruits and vegetables, thereby exacerbating global micronutrient deficiencies [7]. This evolving landscape intersects with heightened consumer demand for healthier foods, fueled by health-consciousness trends and globalized food markets [8,9]. Consequently, advancing food quality analysis technologies has become imperative, both as a safeguard for public health and as a catalyst for industrial innovation, demanding urgent modernization of detection capabilities to address these interconnected challenges [10].

Starting from the source of agricultural food production and raw material acquisition, extreme weather events (floods, droughts, and heatwaves) intensify the spread of foodborne pathogens (e.g., *Salmonella*) and mycotoxin contamination (e.g., aflatoxins) in crops, and ocean acidification increases the risk of algal toxin accumulation (e.g., ciguatoxins) in seafood [11,12]. Pesticide residues (e.g., chlorpyrifos in corn cultivation), veterinary drug residues (e.g., chloramphenicol in meat), alongside heavy metal enrichment (e.g., lead, cadmium) caused by excessive fertilizer use and soil/water pollution, severely degrade crop quality [13,14,15,16].

In processing and production, additives such as cysteine and xylan may enhance fibrous structures in meat analogues [17]. However, excessive or illegal use of processing agents leads to safety failures, for instance, the overuse of butylated hydroxytoluene (BHT) in edible oils [18], or illicitly adding clenbuterol, formaldehyde, borax, nitrites, or nitrates to beef, lamb, and marinated meat products [19]. Although nitrites and nitrates are commonly used as curing agents to inhibit microbial growth, develop characteristic color, and improve flavor, their reaction with secondary amines can generate carcinogenic N-nitroso compounds, raising significant health concerns. Profit-driven adulteration further compromises quality assurance, such as blending soybean protein into chicken mince [20]. During sales, fraudulent practices like falsified origins and nutrient mislabeling necessitate rigorous food classification and compositional testing (e.g., tea grade standards [21] and soluble solids content analysis in cherries [22]). Cold chain disruptions cause secondary contamination, accelerating spoilage, waste, and consumer distrust [23].

Food quality inspection has become imperative for addressing challenges in modern food supply chains. Current analytical technologies encompass spectroscopic analysis, chromatography-mass spectrometry, biosensing and immunological methods, molecular biological techniques, acoustic detection, and electrochemical analysis. Among these, spectroscopic techniques have emerged as the cornerstone of food component analysis and quality control due to their non-destructive nature, multi-component identification capability, and rapid response characteristics. Methodologies exploit interactions between food matrices and electromagnetic radiation, such as absorption, reflection, transmission, scattering, and luminescence (including fluorescence and phosphorescence), to derive molecular and atomic structural information without altering the sample [24]. In molecular spectroscopy, ultraviolet–visible (UV–Vis) spectroscopy identifies adulterants by analyzing absorbance variations induced by electronic transitions. This technique is commonly used to detect adulterants in matrices such as food, pharmaceuticals, cosmetics, and chemical raw materials. Examples include Sudan Red and melamine in food, illegal fillers in pharmaceuticals, prohibited heavy metals, and industrial dyes in cosmetics, as well as low-quality substitute components in chemical feedstocks, all harnessing the characteristic absorption properties of substances within the UV–Vis spectral region for detection [25]. Near-infrared (NIR) spectroscopy leverages overtone/combination vibrations of hydrogen-containing groups (-OH, -NH, and -CH), correlating transmittance/reflectance changes with compositional parameters. This approach demonstrates environmental friendliness through reduced reagent consumption, high-throughput capability, and unique potential for machine learning-assisted complex classification tasks [26,27,28]. Raman spectroscopy employs molecular vibration-induced frequency shifts (Raman displacements), with advanced implementations including surface-enhanced Raman scattering (SERS), spatially offset Raman spectroscopy (SORS), coherent anti-Stokes Raman scattering (CARS), and stimulated Raman scattering (SRS). This technology demonstrates significantly enhanced detection capabilities for deep tissues and trace-level constituents, enabling rapid screening of trace contaminants and accurate identification of food components [29,30,31,32]. Atomic spectroscopic techniques like laser-induced breakdown spectroscopy (LIBS) utilize plasma emission spectra from laser-ablated samples, permitting the rapid elemental analysis of solid/liquid/gaseous matrices with high spatial resolution (μm scale) and minimal invasiveness [33]. Hyperspectral imaging integrates both spectral and spatial resolution, reconstructing 3D chemical distribution maps through hundreds of contiguous narrow bands (e.g., visible–near infrared hyperspectral imaging applied to internal quality assessment in fruits [34], visible–near infrared hyperspectral imaging applied to honey adulteration identification [35]) enables non-destructive analysis of chemical composition, microbial contamination, and physical properties [36,37,38,39]. While being successfully applied to quality detection, this technique faces challenges including data redundancy, environmental interference susceptibility, and model reproducibility limitations [40,41].

Contemporary food inspection technologies exhibit increasing intelligence and multimodal integration. Machine learning algorithms effectively extract spectral/spatial features, while data fusion strategies combining multiple spectroscopic techniques and hybrid spectral/non-spectral datasets significantly enhance the accuracy of evaluation and its generalizability [42,43,44]. In the field of machine learning, traditional machine learning and deep learning technologies exhibit distinct characteristics. Traditional machine learning demonstrates superior performance in small-sample scenarios. For instance, support vector machine-based food composition analysis achieved an accuracy of 97.14% with merely hundreds of samples, while its model feature weights showed high concordance with physicochemical indicators such as near-infrared spectral characteristic wavelengths. This strong interpretability renders it indispensable for regulatory auditing scenarios [45]. However, its heavy reliance on manual feature engineering results in low development efficiency, significant performance degradation when processing the high-dimensional multimodal data, and inadequate modeling capacity for complex nonlinear relationships, constituting pivotal bottlenecks that constrain its advancement [46]. Deep learning overcomes the dimensionality constraints and feature engineering dependency of traditional methods through automated feature processing [47]. Models including convolutional neural networks (CNNs), Recurrent Neural Networks (RNNs), and residual networks (ResNet) exhibit seminal breakthroughs in feature extraction, noise reduction, and nonlinear modeling, substantially enhancing accuracy in both qualitative classification (e.g., variety identification and geographical origin tracing) and quantitative analysis (e.g., component prediction) [48]. Nevertheless, this technology confronts persistent challenges, including data annotation difficulties, limited model generalizability, and excessive computational resource demands [49].

The convergence of spectroscopic technologies and deep learning has provided a rich feature repository, enabling this integrated approach to transcend the environmental parameter limitations inherent in conventional models [50]. By employing CNNs to analyze spectral data from NIR and Fourier-transform infrared (FTIR) spectroscopy, this methodology achieves an accuracy of 90–97% in maturity classification and component quantification for fruits (apples and bananas), as well as quality monitoring of dairy products (milk, cheese), while demonstrating substantially enhanced model interpretability [51,52,53]. The implementation of lightweight architectures (e.g., 4 MB-scale MobileNetv3) coupled with miniature spectrometers enables rapid on-site detection, effectively reducing industrial inspection costs. Meanwhile, the strategic integration of complementary data modalities further improves the system’s generalizability. Models that rely on conventional sensors and manual data collection often fail to capture internal physiological changes [54]. In contrast, hyperspectral imaging technology can detect early physiological anomalies in crops by acquiring spectral data across the 400–2500 nm wavelength range. Combining hyperspectral imaging-derived plant physiological data with environmental parameters optimizes disease prediction models through multidimensional information synergy. The fusion of high-resolution mass spectrometry (HRMS)-acquired metabolic fingerprints with spectroscopic techniques, such as FTIR and NMR, strengthens chemical characterization capabilities for complex samples [55]. Emerging technological syntheses incorporating electronic nose systems, textural features, and classical physical models (Lambert’s cosine law and inverse-square law) present novel optimization pathways for spectral food quality assessment [56,57,58,59].

In the field of food quality detection, numerous studies have systematically reviewed technologies and application scenarios specific to this domain. For instance, consumer-oriented intelligent dynamic detection technologies, such as pH sensors, gas sensors, and biosensors, have been employed for real-time monitoring of freshness, microbial load, additives, and pesticide residues in fresh foods including fruits, vegetables, meat, and aquatic products [10], while electronic nose and electronic tongue systems enable rapid aroma and taste profiling for fruits and vegetables [24]. In aquatic products, near-infrared spectroscopy (NIRS) and near-infrared hyperspectral imaging (NIR-HSI), coupled with chemometric models such as partial least squares (PLS), support vector machines (SVMs), and artificial neural networks (ANNs), have been applied for fish species authentication, origin traceability, real-time freshness evaluation (e.g., TVB-N, K-value, and microbial spoilage), and assessment of nutritional composition and textural properties [60]. Some works parallel investigate the implementation of emerging technologies in food inspection systems, particularly highlighting artificial intelligence’s transformative potential in agricultural applications [61], machine learning-enhanced efficiency and accuracy in food safety protocols [62,63], multi-optical bioanalysis technologies (MOBAs) [30], deep learning architectures [64], and CNNs for adulteration detection and quality assessment [51,65]. Regarding spectroscopic contributions to food science, substantial research delineates the technical advantages and implementation prospects of visible–near infrared (Vis–NIR) and short-wave infrared (SWIR) hyperspectral imaging [66,67,68], NMR spectroscopy [69,70], fluorescence spectroscopy [71], near-infrared spectroscopy [72], and related spectroscopic modalities in quality control applications. Furthermore, cutting-edge investigations synthesize recent progress in integrating advanced technologies with learning algorithms for food security evaluations, exploring complementary synergies between multivariate data analysis (MVDA) and deep learning [73], hyperspectral-machine learning fusion systems [42,74], and spectral-temporal remote sensing (STRS) coupled with deep neural networks [75]. However, the current literature exhibits a notable gap in the systematic consolidation of breakthrough developments from the past three years, specifically addressing the integration of spectroscopic fusion technologies with deep learning architectures in food quality monitoring systems.

This review synthesizes recent advancements in the integration of deep learning with spectral data technologies for food quality analysis, organized into five principal components. The first section initiates a systematic examination of the academic foundations, current research landscape, and technological implementations in food quality assessment. Section 2 methodically categorizes prevalent deep learning algorithms specifically adapted for food quality applications. Section 3 critically analyzes the modeling framework, combining spectral fusion techniques with deep learning architectures. Section 4 conducts a comprehensive evaluation of cutting-edge developments in food quality inspection, presenting a comparative analysis that delineates both innovative breakthroughs and inherent limitations in existing studies while proposing potential research trajectories. The concluding section synthesizes key findings and perspectives, aiming to establish a structured, multi-level conceptual framework that provides scholars with systematically organized references and highlights critical entry points for innovative investigations. By systematically analyzing deep learning-based methodologies for spectral fusion, spectral–non-spectral fusion, and their latest fusion models in food applications, this review aims to identify critical gaps and propose integrative strategies to enhance methodological rigor and drive innovation in spectroscopic data analysis and food quality assessment.

## 2. Classification and Principles of Deep Learning Algorithms

**Figure 1:**
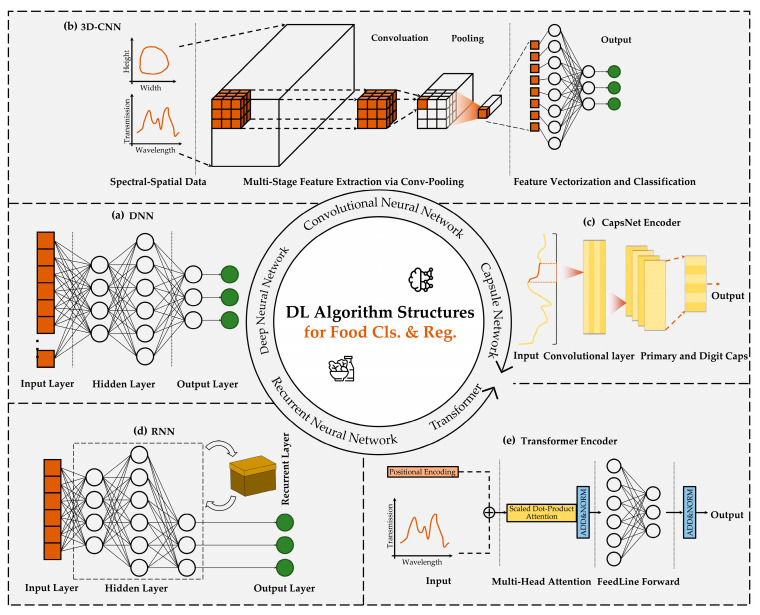
Deep learning network architectures for food classification and regression tasks. (**a**) Deep neural network. (**b**) Three-dimensional convolutional neural network. (**c**) Capsule Network Encoder. (**d**) Recurrent Neural Network. (**e**) Transformer Encoder.

In the domain of food detection, fundamental deep learning algorithms primarily encompass regression, classification, and clustering, supplemented by dimensionality reduction algorithms for preprocessing. Advanced techniques, such as object localization and image segmentation, while crucial for complex applications like real-time production line monitoring, are essentially extensions of these foundational methods. This chapter focuses on core analytical algorithms specifically adapted for food inspection, showcasing their remarkable versatility through strategic modifications to network architectures, loss functions, and training protocols, enabling effective adaptation to diverse task requirements. For regression and classification tasks, established algorithms include deep neural networks (DNNs), CNNs, RNNs, Transformer architectures, and Capsule Networks (CapsNet). Clustering applications typically utilize autoencoders (AEs), variational autoencoders (VAEs), and Deep Embedded Clustering (DEC), with enhanced performance achieved through optimized frameworks like VAE and Stacked Sparse Autoencoders (SSAEs). Emerging hybrid architectures such as CNN–Transformer integrations further demonstrate the field’s evolving methodological sophistication. To provide a comparative overview of the network structures utilized, Figure 1 depicts the architectures of the investigated deep learning models: (a) DNN, (b) 3D CNN, (c) Capsule Network encoder, (d) RNN, and (e) Transformer encoder, each targeting food classification and regression. Table 1 presents the key features, strengths, and limitations, as well as the representative application scenarios, of prototypical deep learning methods applied to regression, classification, and clustering tasks.

### 2.1. Deep Regression and Classification Tasks

The central objective of regression tasks lies in establishing mathematical mappings between input variables and continuous output variables, whereas classification tasks focus on constructing relationships between input variables and discrete categorical labels. When processing instrumental signals, such as spectroscopic data as algorithm inputs, typical preprocessing workflows require sequential steps including noise reduction, baseline correction, and critical feature extraction. These procedures inherently rely on domain-specific chemical knowledge, exemplified by the selective utilization of absorbance measurements at characteristic wavelengths, with compositional concentrations or class labels serving as target outputs. Conventional methodologies, constrained by manual feature engineering and predetermined functional forms, demonstrate limited capacity in processing the high-dimensional, nonlinear relationships. In contrast, deep learning algorithms autonomously construct sophisticated mapping functions through multilayered nonlinear transformations, eliminating the need for explicit mathematical formulations. Neural networks inherently learn hierarchical feature representations through data-driven processes, where shallow layers capture elementary spectral patterns while deeper layers synthesize complex information hierarchies. This distributed representation paradigm effectively addresses the high-dimensional challenges inherent in conventional approaches.

#### 2.1.1. Deep Neural Networks

Deep learning, grounded in the fundamental algorithm of DNNs, employs a deeply layered feedforward architecture expanded from Multilayer Perceptrons (MLPs), extensively applied in food composition regression detection tasks. The DNN structure constitutes a feedforward neural network containing multiple hidden layers, where data undergoes unidirectional transmission from the input layer through successive hidden layer transformations, ultimately generating continuous predictions (e.g., sugar content, fat concentration) at the output layer. In DNN architecture, each neuron establishes full connections with all neurons in adjacent layers, propagating information through weight matrices in a strict feedforward manner without feedback loops. Each hidden layer incorporates nonlinear activation functions (e.g., Rectified Linear Unit, ReLU), enabling hierarchical extraction and combinatorial representation of complex features through multilayer superposition. DNN training relies on the backpropagation algorithm, optimizing network parameters via gradient descent to minimize prediction errors. Compared with shallow networks, DNN’s core advantage in food identification lies in their deep feature extraction capability. Increased layer depth helps capture higher-order nonlinear relationships, making it particularly effective for processing high-dimensional, unstructured data patterns (e.g., spectral or image data). However, its training demands large-scale annotated datasets to prevent overfitting caused by excessive parameters.

#### 2.1.2. Convolutional Neural Networks

Convolutional neural networks (CNNs) represent a specialized class of feedforward neural networks designed for processing spatially structured data. A typical architecture comprises input layers, convolutional layers, pooling layers, and fully connected layers. The input layer accepts one-dimensional/high-dimensional data (e.g., 1D spectra, food surface images, and hyperspectral cubes), with the following convolutional layers extracting local spatial features (e.g., texture patterns and color distributions). Pooling layers reduce feature dimensionality while enhancing translational invariance, leading to continuous value predictions (e.g., moisture and fat content) through fully connected layers. CNNs employ local connectivity and weight-sharing mechanisms to process spatial information, thus effectively minimizing parameter complexity. The network automatically learns local features of food images (e.g., textures and shapes) through convolutional kernels, subsequently combining these features in deeper layers to form global semantic representations. ReLU activation functions are typically employed to enhance nonlinear modeling capabilities, coupled with backpropagation algorithms for parameter optimization [76].

Compared with traditional machine learning methods, CNNs demonstrate significant advantages: Their convolutional architecture enables automatic extraction of hierarchical features, circumventing limitations of manual feature engineering; Pooling operations achieve feature dimension reduction and spatial invariance, exhibiting strong robustness against illumination variations and positional shifts in food imagery; Particularly suited for high-dimensional image processing, they demonstrate superior performance in regression tasks such as foreign object detection and compositional analysis in food science. However, CNN models require extensive annotated datasets for training and substantial computational resources. Increasing network depth may induce vanishing gradient issues, necessitating architectural innovations, like residual connections, for mitigation.

#### 2.1.3. Recurrent Neural Networks

Recurrent Neural Networks (RNNs), along with their enhanced variants, Long Short-Term Memory (LSTM) and Gated Recurrent Units (GRUs), represent a distinct class of deep learning architectures specifically designed for temporal sequence data processing and dynamic system modeling [75]. RNNs establish temporal feature extraction mechanisms through recurrently connected neurons, with LSTM introducing sophisticated gated structures (input gate, forget gate, and output gate) and GRU employing simplified computational frameworks through update and reset gates. These models process data through sequential inputs, with hidden states propagating across time steps to capture dependencies over time.

In spectral food detection applications, RNN-based models demonstrate particular efficacy in fermentation process monitoring, spectroscopic time-series analysis, and continuous sensor data processing from production lines by exploiting sequential relationships where absorption peaks of specific chemical bonds influence multiple consecutive wavelengths [77]. The inherent recurrent architecture enables automatic temporal feature extraction without manual sliding window construction, while the gating mechanisms effectively mitigate the gradual dilution of early-stage information characteristic of traditional RNNs. This capability proves critical for capturing long-range dependencies in dynamic scenarios, such as cold chain logistics temperature forecasting and foodborne disease risk prediction, particularly excelling in multivariate time-series analysis [78]. However, these models present implementation challenges: computational complexity escalates significantly with sequence length, demanding substantial hardware resources; LSTM’s parameter redundancy may induce overfitting in small-sample food datasets, while GRU’s parameter reduction risks compromising temporal detail resolution. Practical implementations require careful architectural selection based on task-specific requirements, coupled with rigorous preprocessing, including temporal alignment of sensor data and appropriate missing value imputation strategies.

#### 2.1.4. Transformer

The Transformer, a deep learning model based on self-attention mechanisms, addresses the inherent limitation of traditional RNNs in parallel computation. In recent years, it has been frequently employed as an embedding module for capturing critical feature information in food flavor regression and classification tasks [79]. Its core architecture primarily consists of stacked encoder and decoder components, though standalone encoder structures are commonly utilized for classification and regression tasks, with decoders typically introduced only when component content quantification is required. Data undergo transformation into high-dimensional vectors through embedding layers, with positional encoding subsequently injecting sequential information. Global feature correlations are captured through multi-head self-attention mechanisms, followed by final prediction outputs through feedforward neural networks. In the attention mechanism, Q (Query), K (Key), and V (Value) stand for the query, key, and value vectors, respectively. Query reflects the current token’s information demand, Key provides features for similarity matching, and Value carries the actual content to be aggregated via attention weights. The attention mechanism in Transformers is mathematically formulated as follows:

(1) $$\mathrm{Attention}(Q,\;K,\;V)=\mathrm{softmax}\;(\frac{QK^{T}}{\sqrt{d_{k}}})\;V$$

Within the Transformer architecture, input vectors at each position dynamically adjust their associations with other positions through attention weights. The model ensures training stability through residual connections and layer normalization while capturing complex feature interactions with position-sensitive nonlinear transformations. Training typically employs the Adam optimizer combined with backpropagation algorithms, enabling accelerated parallel computation. Compared to traditional neural networks, Transformers exhibit superior capabilities in modeling long-range dependencies and demonstrate enhanced representation of complex relationships among the high-dimensional features. The self-attention mechanism’s automatic analysis of inter-wavelength correlations proves particularly advantageous for nonlinear modeling scenarios such as food chemical component analysis. However, these models require substantial training data and face challenges in interpretability, while potentially encountering significant computational complexity when processing the high-dimensional features.

#### 2.1.5. Capsule Networks

Capsule Networks (CapsNets) represent a novel neural architecture employing dynamic routing mechanisms, demonstrating unique advantages in food regression and classification tasks [80]. This framework comprises primary capsule layers (PrimaryCaps) and digit capsule layers (DigitCaps), utilizing vectorized capsule units to replace scalar neurons in conventional neural networks. Data transmission between capsule layers occurs through iterative dynamic routing protocols rather than simple feedforward connections. Each capsule, composed of neuron groups, simultaneously encodes spatial attributes such as feature pose and texture, with vector output magnitudes representing feature existence probabilities.

CapsNet employs nonlinear squashing functions to process capsule outputs, achieving normalization while preserving spatial relationships among features. The training procedure integrates dynamic routing algorithms with backpropagation, optimized through the maximization of correct-class capsule magnitudes. Compared with conventional neural networks, CapsNet’s vectorized capsules and dynamic routing mechanisms enable more effective capture of multi-level spatial correlations in food characteristics (e.g., shape gradations and ingredient distributions), demonstrating enhanced robustness against illumination variations and angular displacements. However, its computational complexity remains substantially higher than traditional fully connected networks, requiring significantly greater hardware resources.

### 2.2. Clustering Tasks

The essence of clustering lies in discovering latent group associations among samples through inherent data structures, independent of predefined class labels. Its input variables encompass instrumental signals such as spectral data, which undergo preprocessing including noise-reduction filtering and baseline calibration, followed by the extraction of chemically representative features such as peak shape similarity in specific wavelength bands or functional group vibrational modes. Traditional approaches rely on manually defined distance metrics and category quantities, whereas classical algorithms, like K-means clustering, require preset group numbers, and hierarchical clustering necessitates artificial selection of dendrogram cutting thresholds. These strong assumptions often lead to suboptimal partitions due to subjective parameter influences when processing the high-dimensional nonlinear distributions characteristic of spectral data. In contrast, deep partitioning algorithms employ autoencoders to learn the low-dimensional embedded spaces, leveraging neural networks’ capacity for modeling complex relationships to automatically amplify intra-class similarities and enhance inter-class distinctions within feature representations. Advanced frameworks, like variational autoencoders and deep embedding grouping networks, further enable the simultaneous optimization of feature encoding and cluster assignment. By preserving chemical characteristics in spectral features while dynamically adjusting set boundaries and morphologies, these approaches effectively resolve traditional methods’ limitations in sensitivity to data distribution patterns and the inability to capture multiscale partitioning structures.

#### 2.2.1. Autoencoder

Autoencoder (AE), a type of unsupervised neural network, is widely employed in food clustering tasks for feature extraction and dimensionality reduction. Its typical architecture comprises an encoder and a decoder. Data are processed through the encoder via feedforward propagation from the input layer, progressively compressed into a low-dimensional latent representation, and subsequently reconstructed through the decoder’s inverse mapping. In AE implementations, encoder neurons typically establish full connections with all nodes in the preceding layer, enabling unidirectional information flow to the latent space. Both encoding and decoding processes utilize nonlinear activation functions to capture intricate structures and nonlinear relationships inherent in food data. During training, AE optimizes parameters by minimizing reconstruction errors between input and output, predominantly through gradient descent algorithms and their variants. Compared with conventional clustering methods, AE demonstrates superior capability in processing the high-dimensional nonlinear food-related data by extracting discriminative low-dimensional features that enhance partitioning accuracy [81]. Nevertheless, AE performance remains sensitive to architectural decisions regarding network depth and latent layer dimensionality, while demanding substantial training data to prevent overfitting and incurring relatively high computational complexity.

#### 2.2.2. Deep Embedded Clustering

Deep Embedded Clustering (DEC) represents an unsupervised methodology that integrates deep feature learning with joint optimization of partitioning objectives. The architecture comprises a stacked autoencoder (SAE) coupled with a grouping layer. The encoder transforms the high-dimensional food data into the low-dimensional latent space through multilayer nonlinear mappings, while the decoder reconstructs the input data, and the grouping layer quantifies sample-to-category similarity using Student’s t-distribution.

In DEC, data undergo hierarchical abstraction through the encoder, with latent space representations and partitioning objectives being co-optimized via a dual-phase strategy. During the pretraining phase, network parameters are initialized by unsupervised minimization of input reconstruction error. The fine-tuning stage refines iteratively group centroid assignments through Kullback–Leibler divergence optimization, while simultaneously adapting the geometric structure of the feature space. This algorithm employs a self-supervision strategy that dynamically generates pseudo-labels through soft assignment probabilities, thereby guiding the network to learn category-discriminative feature representations. However, DEC performance remains highly contingent on pretraining quality and vulnerable to local optima induced by initial group centroid selection. The methodology necessitates careful calibration of weight coefficients between reconstruction and partitioning losses, with computational implementation requiring intensive matrix operations that impose significant hardware resource demands.

## 3. Deep Learning Model and Spectral Data Fusion Techniques

In the context of food quality assessment, fully exploiting the rich chemical and physical information embedded in spectral data necessitates the integration of state-of-the-art deep learning models with multi-source fusion techniques. This chapter begins with an overview of prevalent spectral modalities and their distinctive characteristics, followed by a presentation of modeling strategies tailored to diverse inspection tasks, and concludes with an exploration of concrete approaches for fusing heterogeneous spectral information. By systematically analyzing the interplay between modeling and data fusion, this chapter lays the theoretical groundwork for subsequent application and performance enhancement.

### 3.1. Principles and Characteristics of Spectral Technology

Spectral techniques identify chemical components based on optical responses (e.g., absorption and reflection) at different wavelengths, offering non-destructive, rapid, and sensitive analysis. Each method has unique strengths in terms of spectral range and detection capabilities. This section elucidates the fundamental principles and representative application scenarios of near-infrared/mid-infrared spectroscopy, Raman spectroscopy, hyperspectral imaging (spanning the UV, visible, and NIR regions to simultaneously capture both spatial distribution and spectral signatures of sample constituents), fluorescence spectroscopy, terahertz spectroscopy, and nuclear magnetic resonance spectroscopy, highlighting their critical properties and suitability for various food inspection tasks. Table 2 provides a detailed overview of the practical applications of various spectroscopic techniques in food quality assessment, summarizing each of the employed models, study subjects, and spectral feature types.

**Table 2:** Summary of different spectral technologies applied for food quality detection.

Techniques	Samples	Applications	Spectral Feature Type	Methods	Reference
NIR	Citrus	Prediction of sugar content	MSC, SNV (472–1156 nm)	ENM	[82]
	Citrus	Prediction of SSC and vitamin C content	MSC, SNV (560–1000 nm)	CNN	[83]
	Crown pear	Prediction of SSC	SG, SNV (610–960 nm)	MLP-CNN-TCN	[84]
	Durian	Prediction of nutritional components	SNV, 2nd Der (860–1760 nm)	DNN	[85]
	Ligusticum chuanxiong	Identification of geographic origin	SG, MSC (e.g., 2439–2500 nm)	CNN	[86]
	Matsutake	Identification of geographic origin	SNV (900–1700 nm)	CNN	[87]
	Oil palm	Prediction of free fatty acid content	Higuchi fractal dimension (1000–1500 nm)	LSTM and GRU	[88]
	Panax quinquefolius	Identification of geographic origin	SNV, min-max scaling (900–1650 nm)	AGOTNet	[89]
	Perilla	Prediction of mineral content in seeds	SNV, min-max scaling (400–2500 nm)	SVR, ANN, RFR, and CNN	[90]
	Porphyra yezoensis	Detection of phycobiliproteins	SNV, 1st Der (900–1650 nm)	CNN	[91]
NIR	Potato	Prediction of starch content	SNV, 1st Der (940–1650 nm)	CNN	[92]
	Procambarus clarkii	Assessment of multiple freshness	e.g., SG, SNV (940–2500 nm)	CNN	[93]
	Vine tea	Detection of tps and dmy content	SNV, 1st Der, 2nd Der (900–1700 nm)	CNN-LSTM	[94]
	Watermelon	Prediction of SSC	Mean Centralization (900–1700 nm)	CNN	[52]
RS	Corn oil	Quantification of ochratoxin	SG (20–2000 cm^−1^, 737–1455 cm^−1^)	CNNs, PLSR, RFR, and GPR	[95]
	Dairy products	Classification of species	Normalization, specific ranges (e.g., 890–980 cm^−1^)	SVM, ELM, and CNN	[96]
	Edible oils	Prediction of antioxidants	SG, air-PLS (1200–1800 cm^−1^)	CNN	[97]
	Green tea	Identification and classification of acetamiprid and thiacloprid residues	SG, air-PLS (300–2000 cm^−1^)	CNN, BP, and AlexNet	[98]
	Pork	Prediction of gel strength and whiteness in pork paste	Labspec 6 (400–3200 cm^−1^)	CNN-LSTM	[99]
HSI	Bok choy	Identification of pests	NDVI, PPC (e.g., 420–440 nm)	DNN	[100]
	Chicken	Classification of blood-related defects in the chicken’s chest	IFBA (e.g., 420–600 nm, 950–970 nm)	CNN	[101]
	Coriander	Classification and prediction of low-temperature damage	Bilinear downsampling, Bayesian wavelet denoising, median filtering (900–1700 nm)	CNN	[102]
	Corn seeds	Identification of freezing damage on the embryo and endosperm sides	SNV (450–979 nm)	DCNN	[103]
	Eggs	Detection of cracks, dirt, and blood spots	Otsu’s thresholding method (400–1000 nm, 690–780 nm)	DNN and CNN	[104]
	Maize	Distinction of corn kernels	Orthogonal signal correction (e.g., 935–990 nm)	CNN	[105]
	Maize	Identification of fungal species	WDR, min-max Norm. (996–2501 nm)	MCRM-CNN	[106]
	Ophiopogonis radix	Identification of geographic origin	WDR, SG (400–1000 nm)	M3DC-Transformer	[107]
HSI	Peanuts	Classification of aflatoxin contamination	PCA (292–865 nm, 400–2500 nm)	CNN	[108]
	Peanuts	Detection of aflatoxin B1	ConvAE (415–799 nm)	LSTM	[109]
	Potato	Prediction of anthocyanin content	SG, SNV, detrending (365–1025 nm)	CNN	[110]
	Red meat	Prediction of PUFA content	Raw spectrum (400–1000 nm)	AE-GAN	[81]
	Rice seeds	Prediction of anthocyanin content	SG, 1st Der, 2nd Der (425–1690 nm)	DCGAN and CNN	[111]
	Salmon	Identification of geographic origin	SG, WDR (400–1000 nm)	CNN-BiGRU	[112]
	Sorghum	Prediction of sorghum protein content and moisture content	Raw spectrum (886–1735.34 nm)	CLNet	[113]
	Soybean	Classification of lodging rating and soybean yield forecast	WDR (450–950 nm)	PCL	[114]
	Strawberry	Classification and identification of strawberry ripeness	WDR, sequential feature selection algorithm (370–1015 nm)	SVM and CNN	[115]
	Surimi	Prediction of multiple quality indicators	WDR, 1st Der, 2nd Der (400–1700 nm)	CNN-LSTM	[116]
	Sweet potato	Prediction of SSC	WDR, MSC, SNV, SG (400–1000 nm)	CNN	[117]
	Wheat	Identification of species	WDR (397–1004 nm)	DLFM	[118]
FS	Almonds	Classification of aflatoxin contamination	Image cropping, color space (375 nm, 435 nm)	CNN	[119]
	Dark tea	Classification of brands and aging periods	Background and scatter correction (230–530 nm, 244.73–827.81 nm)	CNN	[120]
	Olive oil	Prediction of five chemical quality indicators	Background subtraction, normalization (650–750 nm, 500–800 nm)	CNN	[121]
THz	Rice seedlings	Prediction of nitrogen content in roots	CLAHE, PCA (0.1–3.5 THz)	CNN, GA-BPNN, and SSA-SVR	[122]
	Sunflower seeds	Identification and classification of seeds	Background subtraction, normalization (0.1–3 THz)	CNN-Transformer	[123]
	Wheat	Classification of wheat varieties	SNV (0.2–1 THz)	CNN	[124]
NMR	Honey	Identification of adulterant sugars	PH adjustment and internal standard addition (5.3–5.5 ppm)	LR, DNN, and LGBM	[125]

Abbreviations: ENM, Ensemble neural network method; CNN, Convolutional Neural Network; DNN, Deep Neural Network; DCNN, Deep Convolutional Neural Network; MLP, Multi-Layer Perceptron; TCN, Temporal Convolutional Network; PCL, Prototype Contrastive Learning; SG, Savitzky-Golay; WDR, White/dark correction; CLNet, a fusion model combining a one-dimensional Convolutional Neural Network and a Long Short-Term Memory network; tps, tea polyphenols; dmy, dihydromyricetin; SSC, Soluble Solid Content; FUFA, polyunsaturated fatty acid; PLSR, Partial Least Squares Regression; RFR, Random Forest Regression; NDVI, Normalized Difference Vegetation Index; PPC, Percentage Point Change; IFBA, Improved Firefly Algorithm; MSC, Multiplicative Scatter Correction; GPR, Gaussian Process Regression; AE-GAN, autoencoder-assisted generative adversarial network; M3DC-Transformer, **multi-scale 3D convolution** and **Transformer; DLFM,** dual-channel deep learning feature fusion model; ConvAE, Convolutional autoencoder; MCRM-CNN, Multi-Channel Residual Module Convolutional Neural Network; LGBM, Light Gradient Boosting Machine; AGOTNet, External Attention Guided Origin Traceability Network; LSTM, Long Short-Term Memory; SVM, Support Vector Machines; BiGRU, Bidirectional Gated Recurrent Unit; GA-BPNN, Genetic Algorithm-Back Propagation Neural Network; SSA-SVR, Sparrow Search Algorithm-Support Vector Regression; CLAHE, Contrast Limited Adaptive Histogram Equalization.

#### 3.1.1. Near-Infrared/Mid-Infrared Spectroscopy

Near-infrared spectroscopy (NIRS) exploits overtone and combination band absorption arising from molecular vibrations, capturing vibrational characteristics of hydrogen-containing groups (O-H, N-H, and C-H) through various optical configurations such as diffuse reflectance, specular reflection, transmittance, and directional transmittance modes, as shown in the following Table 3. By correlating these spectral features with target components, predictive models are established, enabling rapid analysis of major food constituents such as moisture, protein, and fat. Its non-destructive nature and substantial sample penetration capacity make it particularly suitable for online monitoring of solid or powdered food products like cereals [126,127]. Deep learning algorithms autonomously extract nonlinear spectral features, optimizing model generalization capability to enhance prediction accuracy in complex food matrices. For example, Huang et al. [83] used NIRS and a one-dimensional CNN to predict soluble solids content and vitamin C in Citrus reticulata ‘Ai Yuan 38′. Mid-infrared spectroscopy (MIRS) directly resolves fundamental vibrational modes of chemical bonds (C=O, O-H) through characteristic absorption peaks, demonstrating superior chemical specificity. The implementation of attenuated total reflection (ATR) technology streamlines the analysis of viscous or semi-solid samples, enabling precise identification of molecular transformations such as carbohydrate isomerization [128]. However, stringent requirements for sample transparency restrict its application in turbid systems, typically necessitating sophisticated instrumentation for offline analysis.

#### 3.1.2. Raman Spectroscopy

Raman spectroscopy (RS) operates on the principle of inelastic scattering, acquiring chemical bond vibrational information through analysis of frequency shifts caused by interactions between incident light and molecular vibrations. This technique demonstrates superior sensitivity to nonpolar bonds, such as C–C and S–S, as well as symmetric molecular structures, while exhibiting minimal interference from water molecules. These characteristics render it particularly suitable for non-destructive analysis of the high-moisture food products, including beverages and fresh agricultural produce. Surface-enhanced Raman spectroscopy, when integrated with deep learning-driven feature extraction, achieves significant signal amplification through nanoscale substrate engineering, enabling on-site rapid screening of trace-level pesticide residues and mycotoxins [29]. For instance, highly anisotropic silver nanoparticles (AgNPs) synthesized via response surface methodology exhibited an exceptional enhancement factor (EF) of ~10^8^, facilitating the detection of neonicotinoid pesticides (acetamiprid, ACE; thiacloprid, THI) in green tea down to concentrations as low as 10^−7^ mg/mL [98]. Combining HSI with surface-enhanced Raman spectroscopy (SERS) unites macro-scale component imaging and micro-scale molecular fingerprint analysis, and by fusing multiscale information through multimodal deep models, it further enhances the sensitivity for detecting food safety indicators (e.g., pesticide residues and mycotoxins), enabling rapid, accurate, and non-destructive online screening of high-moisture food products such as beverages and fresh produce.

#### 3.1.3. Hyperspectral Imaging

Hyperspectral imaging (HSI) technology integrates spectroscopic analysis with spatial imaging capabilities, acquiring continuous spectral bands on a pixel-by-pixel basis to simultaneously resolve both chemical composition distribution and physical morphological characteristics of food products [129,130]. The short-wave near-infrared (SW-NIR) spectral regime enables subsurface detection of meat tenderness and identification of mold-contaminated regions in grains, while the visible spectral range proves particularly effective for analyzing surface color variations and detecting defects in fruits and vegetables. Deep learning frameworks demonstrate exceptional proficiency in processing multidimensional Vis–NIR hyperspectral datasets, enabling precise extraction of diagnostic spectral wavelengths and construction of robust classification models. Gao et al. [115] demonstrated the real-time, in-field estimation of strawberry ripeness using a portable HSI system. Key visible wavelengths identified via sequential feature selection enabled high-precision classification of ripeness stages using support vector machines. Furthermore, deep learning (AlexNet CNN) achieved high accuracy (98.6%) by effectively fusing spatial-spectral features from key wavelengths and principal components. This technical framework has been successfully implemented in automated production lines for non-destructive quality assessment, achieving real-time monitoring of agricultural product integrity [42,131,132,133].

#### 3.1.4. Fluorescence Spectroscopy

Fluorescence spectroscopy (FS) achieves exceptional sensitivity in detecting compounds containing conjugated double bonds or aromatic rings, such as vitamin B2, chlorophyll, and polycyclic aromatic hydrocarbons (PAHs), by monitoring electronic energy level transitions and capturing characteristic emission signals [67,134,135]. As an illustration, excitation-emission matrix (EEM) fluorescence spectroscopy coupled with convolutional neural networks (CNNs) has been effectively employed to identify dark tea brands and determine their aging periods based on the distinct fluorescent profiles of key components like fulvic-like substances and theabrownin [120]. Time-resolved fluorescence technology enhances analytical specificity through pulsed excitation that discriminates short-lived autofluorescence from long-lived target emissions, enabling precise quantification of trace toxins in complex matrices. When integrated with sequential modeling algorithms, this approach permits dynamic monitoring of adenosine triphosphate (ATP) degradation products in aquatic products and real-time assessment of quality deterioration during fruit and vegetable storage. The technique’s primary limitations arise from its dependence on target-specific fluorescent properties and potential spectral interference from the high-concentration pigments.

#### 3.1.5. Terahertz Spectroscopy and Nuclear Magnetic Resonance Spectroscopy

Terahertz spectroscopy (THz) employs low-frequency photons to excite weak intermolecular interactions, such as hydrogen bonding and lattice vibrations, showing exceptional sensitivity to rotational transitions of polar molecules (e.g., water) and polymorphic transitions in crystalline materials. For example, THz imaging combined with the MobileViT-E model achieved 96.30% accuracy in classifying damaged and deformed sunflower seeds by capturing internal structural features invisible to conventional methods [123]. Terahertz imaging technology enables root phenotyping extraction and quantitative nitrogen analysis through the synchronous acquisition of two-dimensional spatial images coupled with one-dimensional spectral data [122]. Nuclear magnetic resonance (NMR) spectroscopy operates on the principle of nuclear spin transitions in magnetic fields, where chemical shifts reveal molecular electronic environments, while relaxation times characterize molecular dynamics. Notably, NMR identified honey adulterated with sugar syrups (e.g., corn, rice, jaggery) at 5–30% concentrations with 100% accuracy via machine learning classification of oligosaccharide fingerprints in the 5.3–5.5 ppm spectral region [125]. This technique offers distinct advantages, including high reproducibility, non-destructiveness, and minimal sample preparation requirements, while maintaining the capability for simultaneous detection of both polar and non-polar metabolites. However, its limitations encompass lower sensitivity compared to mass spectrometry and high instrument costs [69].

### 3.2. Modeling Methods

In food inspection tasks, raw spectral data are often high-dimensional and redundant, requiring appropriate modeling strategies to enhance generalization and robustness. Deep learning frameworks provide superior capabilities for feature extraction and nonlinear representation; however, their architecture design and training protocols must be tailored to the characteristics of spectral data. This section focuses on two representative strategies: modularization and phased-fusion modeling. The former improves model interpretability and flexibility by decomposing tasks into functional submodules, while the latter leverages the synergy of multi-stage learning and feature fusion, particularly effective for processing complex or multi-source spectral inputs. These approaches lay the groundwork for efficient and scalable spectral data fusion in downstream applications. The deep learning models referenced in this section are summarized in Table 4.

#### 3.2.1. Modularization

Modularization refers to the design strategy of constructing deep neural networks using interchangeable and reusable components, or modules, such as convolutional blocks, attention units, or residual structures. These modules can be independently developed, optimized, and combined to form more complex architectures, thereby enhancing the flexibility, interpretability, and scalability of the model design.

The widespread application of deep learning in spectral analysis has made the efficient capture of multi-granularity features a central challenge in model design. Traditional CNNs employing single-sized kernels struggle to balance local details with global patterns, often leading to critical spectral feature loss or computational redundancy. To address this bottleneck, Szegedy’s team [136,137] drew inspiration from the multi-receptive field mechanisms in biological vision systems, innovatively designing the Inception module architecture. By integrating Inception modules with residual connections, they proposed the Inception-ResNet series. This architecture synergistically combines parallel multi-scale feature extraction pathways through coordinated integration of 1 × 3 spectral convolution, 3 × 5 cross-band filtering, and max-pooling operations. Subsequent channel-wise compression enables deep feature fusion, significantly enhancing spectral feature representation capabilities. Zhang et al. [138] further optimized the CNNs architecture by combining modified Inception modules with attention mechanism modules, accomplishing the first successful implementation of simultaneous internal and external quality detection in Nanfeng tangerines. The standalone incorporation of Inception blocks expands network width, enabling concurrent learning of diverse feature representations within the same layer. This architectural enhancement enriches model comprehension of input data and markedly improves both qualitative and quantitative prediction performance. Furthermore, in non-invasive starch content prediction for potato tubers using near-infrared spectroscopy, the InceptionV3 model exhibited significant superiority over conventional models, including robust linear regression (RLR), PLSR, Lasso, and principal component regression (PCR) [92].

In the modeling of hyperspectral data within complex scenarios, the efficacy of multi-scale feature fusion critically influences recognition accuracy, emerging as a pivotal bottleneck constraining model performance. Conventional hierarchical network architectures exhibit inherent limitations in harmonizing shallow-layer detailed information with deep semantic features, often suffering from information attenuation and compromised equilibrium between local responses and global contextual relationships. The feature adaptive fusion module has consequently emerged as an effective solution, preserving original feature dimensions while incorporating constraints on feature importance coefficients. This innovation enables dynamic weighting of spectral and image characteristics within fused information. When integrated with CNN-extracted outputs, this adaptive fusion approach demonstrates remarkable classification enhancement, achieving an accuracy of 99.18% in wheat cultivar identification [118]. Similarly, the Spectral-Image Feature Fusion Convolutional Neural Network (S-IFCNN) for geographical origin authentication of wolfberries leverages this adaptive fusion mechanism to attain an accuracy of 91.99% on the test set [139].

Traditional neural networks often suffer from weakened discriminative attributes in critical spectral bands due to global feature coupling effects when processing the high-dimensional spectral data. This performance degradation becomes particularly pronounced under conditions of strong background noise or spectral line overlap, where the model’s capacity to detect subtle spectral variations is significantly impaired. The Squeeze-and-Excitation (SE) module pioneers a channel self-calibration paradigm by establishing a dynamic weight modulation system that enables intelligent feature channel selection [140]. This mechanism, designed based on feature recalibration theory, combines global information embedded with gating functions through a two-stage process: first, compressing spatial dimensions to obtain global statistics for each channel, then learning nonlinear interdependencies between channels via bottleneck structures, ultimately generating channel attention weights with inherent spectroscopic significance. For meat freshness detection, Cheng et al. [141] developed a Hybrid Fusion Attention Network (HFA-Net) that synergistically integrates early and late fusion architectures in parallel. This design effectively captures cross-modal interactions while employing SE modules to focus on critical features, thereby circumventing the curse of dimensionality.

While increasing network depth enhances feature abstraction capabilities, it progressively reveals the challenges of gradient attenuation and shallow-layer feature loss. The multi-channel residual module (MCRM) proposed by Long’s team addresses these issues through three sets of parallel residual blocks that enable multi-scale feature fusion, complemented by shortcut connections to mitigate gradient vanishing [142]. This design preserves the inherent advantages of residual networks while employing heterogeneous convolutional kernels to capture discriminative information from nanometer-scale fluctuations in spectral curves. Experimental results demonstrate that MCRM-CNN achieves a classification accuracy of 94% on the test set for four mycotoxin-producing fungal species (including Aspergillus flavus) versus healthy samples, directly extracting latent features from 366 Raman wavenumbers without relying on manual feature selection.

#### 3.2.2. Phased and Fusion Modeling

Phased modeling in this study refers to a sequential and stage-wise approach to model development, where each phase focuses on a specific sub-task, such as feature extraction, pretraining, fine-tuning, or decision integration. This is distinct from end-to-end training and allows for greater control and interpretability of each modeling step.

In spectroscopic analysis, extracting meaningful chemical information and enhancing model accuracy remain critical challenges in food detection. Beyond modular innovations, model fusion has emerged as a promising solution. Fusion modeling indicates the integration of multiple feature representations or model outputs, either at the feature level (early fusion), intermediate layer level (mid-level fusion), or decision level (late fusion), to enhance model robustness and generalization. The integration of CNN with partial least squares regression (PLSR) and support vector regression (SVR) enables the development of hybrid CNN-PLSR and CNN-SVR architectures. These frameworks leverage CNN’s automated feature extraction from spectral data, followed by PLSR/SVR prediction, demonstrating superior performance compared to conventional PLSR/SVR implementations and standalone CNN models [143]. Xu et al. [144] validated the efficacy of deep feature extraction through a phased fusion approach combining Sparse Autoencoder-derived features with least squares support vector machine (LS-SVM) prediction, achieving accurate quantification of total soluble solids (TSS) and titratable acidity (TA) in Kyoho grapes. Yue et al. [145] addressed spectral autocorrelation and nonlinearity challenges through the synergistic integration of radiative transfer modeling (RTM) with a Lightweight Attention-optimized CNN (LACNet). This innovative fusion significantly enhanced feature extraction capabilities in sensitive spectral regions (particularly red-edge wavelengths), substantially improving estimation accuracy for leaf area index and chlorophyll content in plant systems.

The integration of SVM with deep learning architectures has become increasingly prevalent. Fu’s team [146] developed an SSAE-CS-SVM model combining a stacked sparse autoencoder with Cuckoo Search (CS) optimization for SVM parameter tuning, achieving effective maize seed variety identification. Gao et al. [115] employed hyperspectral imaging (HSI) systems to acquire spectral data, implemented a sequential feature selection (SFS) algorithm for characteristic wavelength screening, and validated it through SVM classification. Furthermore, by applying principal component analysis (PCA) to extract the first three principal component images and utilizing pre-trained AlexNet CNN for classification, they attained a classification accuracy of 98.6% in laboratory sample testing. Regarding quality grading applications, the enhanced fast and precise YOLOv5 (FP-YOLOv5) model combined with the successive projections algorithm (SPA) and PCA demonstrates the classification accuracy of 95% in early bruise recognition for apples at 130 frames per second [106]. Modified ResNet-50 architecture integrated with PCA has shown promising performance in tea quality classification [147].

The integration of multiple deep neural networks has introduced novel solutions for food quality detection. Models combining convolutional neural networks’ feature extraction with Transformer attention mechanisms demonstrated significantly superior prediction accuracy for cherry tomato soluble solid content (SSC) compared to traditional PLSR and SVR methods, with enhanced model interpretability through Grad-CAM visualization [22]. By integrating 1D convolutional neural networks (1D-CNN) with long short-term memory networks (LSTM), Hu et al. [113] achieved non-destructive detection of sorghum protein and moisture content using hyperspectral technology, while Xia et al. [116] implemented quality monitoring of silver cod surimi in NIR bands. Zhang’s team [148] further enabled precise prediction of lily polysaccharides, total phenols, and sulfur dioxide content through variable combination population analysis (VCPA). In geographical traceability, Yi et al. [149] proposed a Tran-MPRNet model integrating a multi-stage progressive image restoration network (MPRNet) with transfer learning, reconstructing visible–near-infrared (Vis–NIR) hyperspectral data from RGB images for beef origin identification.

The stacking ensemble model, an advanced ensemble learning technique, employs a strategic combination of diverse base models to enhance overall predictive performance and generalization capability. Kim et al. [82] proposed an ensemble neural network architecture (Ensemble Type-4) that effectively integrated CNNs with classification sub-models through a modular architecture, demonstrating superior processing of multi-species spectral data. Cheng et al. [150] substantiated that stacking ensemble models achieve enhanced robustness and generalization capacity in food cadmium detection by synergistically combining predictive advantages from multiple base learners (SVR, ELM, DT, RF), thereby establishing a novel approach for foundational model fusion strategies. This methodology demonstrates significant potential for complex analytical applications requiring multi-model collaboration.

### 3.3. Multi-Source Spectral Information Fusion

A single spectral modality often falls short of capturing the multidimensional quality attributes inherent in complex food systems. Multi-source spectral fusion techniques, which integrate various spectral modalities or heterogeneous data (e.g., spectroscopy combined with imaging), can substantially enhance a model’s perception and discrimination capabilities. Fusion strategies can be broadly categorized into spectral fusion, at the band or feature level, and heterogeneous data fusion, across different sensing modalities, enabling complementary information extraction. This section introduces common fusion frameworks and implementation techniques, highlighting their advantages in tasks such as quality prediction and adulteration detection, while laying the theoretical foundation for designing cross-modal deep learning architectures.

#### 3.3.1. Spectral Fusion

In multi-source spectral information fusion, data-level aggregation, feature-level extraction, and decision-level synthesis constitute three hierarchical strategies operating at distinct stages of the data processing pipeline. Among these, data-level aggregation—combining diverse raw spectral datasets—and feature-level extraction—integrating distinct spectral characteristics to capture key patterns—have gained widespread adoption. This section focuses on their application in conjunction with deep learning approaches. Decision-level synthesis, which integrates outputs from individual models through mechanisms such as majority voting, weighted averaging, or meta-classifiers, has demonstrated potential in certain applications but remains less common; thus, it falls outside the primary scope of this review.

Consistent preprocessing of multisensor spectral data is critical for effective fusion. Typical steps include radiometric calibration (e.g., dark current subtraction and standard white reference correction) to convert raw digital counts into calibrated reflectance values. Spectral normalization techniques, such as Standard Normal Variate (SNV) and Multiplicative Scatter Correction (MSC), are applied to correct baseline shifts and scattering effects across spectra. Noise filtering algorithms (e.g., Savitzky–Golay smoothing) are employed to suppress high-frequency sensor noise. These preprocessing procedures help to standardize spectral signatures from different sources and improve the consistency of modeling inputs, thereby enhancing the overall performance and robustness of data fusion models.

Fluorescence Hyperspectral Imaging (F-HSI) represents an innovative solution addressing the spatial resolution limitations inherent in conventional fluorescence detection methodologies. Traditional point-scan fluorescence spectroscopy techniques struggle to comprehensively characterize the spatial heterogeneity of optical properties across sample regions, whereas F-HSI integrates the advantages of fluorescence spectral techniques and hyperspectral imaging to simultaneously acquire both spectral signatures and spatial information for every image pixel [151]. Hao et al. [134] demonstrated the efficacy of this approach through fluorescence hyperspectral imaging coupled with heterogeneous two-dimensional correlation spectral methods (2D-COS) analysis for resolving complex fluorescence peaks, enabling rapid quantification of total polycyclic aromatic hydrocarbons (T-PAHs) in roasted Tan mutton.

NIR spectroscopy is frequently employed in conjunction with adjacent spectral techniques. For instance, Joshi et al. [152] integrated Ultraviolet-Visible-Near Infrared (UV–Vis–NIR) spectroscopic data for strawberry shelf-life monitoring. Pipatsart et al. [102] developed a deep learning approach utilizing short-wave infrared hyperspectral imaging (SWIR-HSI) combined with CNNs, achieving early detection of chilling injury in fresh coriander through spatial-spectral feature fusion. Raman spectral methods have gained substantial traction in food analysis due to their non-destructive nature, requiring minimal sample preparation, and employing inelastic scattering for molecular characterization [153]. Ma et al. [154] comprehensively reviewed Raman-based fusion strategies, demonstrating that dual-modal “Raman plus X” spectroscopy—integrating Raman with complementary techniques (e.g., NIR, NMR, THz, LIBS, and fluorescence)—significantly enhances discriminative capabilities. Notably, David et al. [155] successfully implemented honey classification and geographical traceability by fusing infrared (600–1800 cm^−1^) and Raman (200–1799 cm^−1^) spectral data combined with deep learning algorithms.

LIBS has demonstrated significant potential for quantifying nutritional elements in food products [14]. Chen et al. pioneered a multimodal approach by integrating LIBS with visible and near-infrared (Vis–NIR) optical methods, where LIBS-derived elemental information synergistically combines with Vis–NIR-acquired molecular signatures through deep learning-based feature-level fusion, achieving remarkable enhancement in adulteration detection accuracy for Polygonati Rhizoma [156]. The synchronous and asynchronous 2D-COS features, serving as complementary feature enhancements from identical spectral data, were effectively fused via bilinear pooling to capture spatial interaction patterns between spectral sequences, demonstrating superior performance over single-spectrum analysis [157]. In a groundbreaking study of non-destructive analysis for lipid oxidation in freeze-thawed pork, Cheng’s team successfully identified six characteristic bands through generalized 2D-COS analysis of Vis–NIR spectra. Subsequent heterogeneous 2D-COS correlation between Vis–NIR and fluorescence spectra revealed seven additional feature bands, substantially expanding feature identification capability [158]. Spectral techniques also enable the monitoring and early warning of crop stress states by detecting specific gaseous molecules released by plants, such as ethylene and volatile organic compounds (VOCs), through analyzing the correlations between their metabolic alterations and physiological activities. For instance, Zhang et al. demonstrated the integration of hyperspectral technology (400–1000 nm) with tunable diode laser absorption spectroscopy (TDLAS) gas detection methodology, achieving early identification of heat stress (HS) and UV-B stress (UV-BS) in wheat crops [159].

Furthermore, an innovative information screening strategy involving multi-perspective imaging (lateral and top views) has been developed, where systematic elimination of interference sources (fruit stems and calyxes) enables effective retention of critical lateral view data. This methodology provides valuable insights for noise reduction and fusion optimization in multi-angle/multimodal spectral data analysis [160].

#### 3.3.2. Spectral-Heterogeneous Data Fusion

Multimodal fusion of spectral and non-spectral data (such as hyperspectral images with RGB or NIR imagery) demands careful preprocessing to coordinate across data types. Spatial co-registration aligns images so that pixels from different sensors represent the same ground locations. Scale normalization reconciles differences in spatial resolution and intensity scales between datasets, and pseudo-color or grayscale mapping may be used to project high-dimensional spectral data into comparable channels with conventional images. Region-of-interest (ROI) synchronization or consistent segmentation ensures that subsequent analysis focuses on identical spatial regions in all modalities. By harmonizing both the spatial geometry and the spectral content of each modality, these preprocessing steps enable coherent integration of multimodal information.

Mass spectrometry demonstrates core strengths in molecular weight specificity, high sensitivity, and untargeted analytical capability, effectively addressing the limitations of spectroscopic techniques in complex matrix resolution, trace detection, and structural verification [55]. The synergistic integration of Raman spectroscopic imaging with MALDI-MSI enables comprehensive spatial distribution mapping and structural characterization of food components [161]. Concurrently, LC-MS compensates for the inherent sensitivity constraints of NMR through superior quantification limits and extensive metabolite coverage [162]. Fundamental physical principles further enhance spectroscopic data quality. Han et al. [163] achieved high-precision dynamic monitoring of vegetation water content by coupling spectral models with photon transport mechanisms (PROSPECT-PROSAIL cross-scale simulations), integrating MODTRAN-based atmospheric correction techniques for quantifying transmittance, and fusing multi-source remote sensing data (ground measurements with satellite imagery). Zhu et al. [59] significantly improved robustness against illumination variations and imaging distances in complex canopy systems (e.g., perilla and tea seedlings) through physics-informed spectral corrections based on Lambert’s cosine law and inverse square law.

Cross-modal data fusion strategies further advance analytical precision in food inspection. Cheng et al. [56] markedly enhanced moisture prediction accuracy in freeze-thaw cycled pork by combining hyperspectral imaging with electronic nose data. The integration of colorimetric sensor arrays with near-infrared spectroscopy, coupled with CNN processing, enables rapid quantitative detection of zearalenone (ZEN) contamination in wheat [164].

## 4. Recent Advances in the Integration of Spectral Analysis and Deep Learning

This chapter presents a comprehensive review of the diverse application directions arising from the integration of spectral analysis and deep learning in food inspection. Organized into two major sections—qualitative detection (Section 4.1) and quantitative detection (Section 4.2)—it first examines methodologies for food traceability, adulteration identification, and classification. The quantitative detection section then explores recent advances in predicting heavy metal concentrations, harmful toxins, and nutrient content. Throughout, the focus remains on how network design, loss formulation, and multimodal fusion synergistically enhance accuracy, robustness, and generalization in diverse practical scenarios.

### 4.1. Qualitative Detection

High-precision identification of food varieties plays a pivotal role in quality traceability and market regulation, while the limitations of single-spectral modalities have spurred the rapid development of multi-source fusion technologies. In recent years, data/feature fusion techniques integrated with deep learning have been successfully applied to food qualitative analysis tasks, including flaxseed [79], maize seeds [165], soybean leaves [166], coriander [102], broccoli [89], wheat [118,159], Polygonatum [156], apple [167], honey [155], fish [168], dairy products [96], and others. Qualitative tasks combining spectroscopy with deep learning primarily include food traceability, adulteration identification, and freshness assessment. Food traceability technologies can track or differentiate the origin of food, ensuring the authenticity and safety of its source. Adulteration detection focuses on identifying illicit ingredients or substandard products within food, protecting consumer rights. Freshness assessment technologies are used to evaluate the quality and freshness of food, ensuring that it reaches consumers in optimal condition. The application of these technologies provides effective assurance for food safety.

#### 4.1.1. Food Traceability Detection

The combination of HSI and deep learning technologies has been widely applied in food origin tracking. Addressing the issue of origin identification of Pu-erh ripe tea in the tea market, Chen et al. [169] proposed a method combining HSI with CNN to identify the origin of Pu-erh ripe tea. The recognition accuracy of the CNN model reached 95.66%, outperforming traditional models such as SVM and Partial Least Squares Discriminant Analysis (PLS-DA). Goji berries, as a commonly used medicinal herb and edible plant, have their price and quality largely dependent on their origin. To effectively trace the origin of goji berries, Jiang et al. [139] proposed a method combining HSI and S-IFCNN. The study showed that the S-IFCNN model, by fusing one-dimensional spectral and two-dimensional image data, could accurately distinguish goji berries from different origins, with a classification accuracy of 91.99%. Salmon, being rich in high-quality proteins and healthy fatty acids, has become a globally popular nutritious food. Zou et al. [112] utilized HSI and an improved CNN-BiGRU model to identify the origin of salmon, achieving an accuracy of 99.5% on the test set. FTIR combined with deep learning methods has also been widely applied in the traceability of medicinal plants. Deng et al. [170] proposed a rapid method for identifying the origin of Gastrodia elata, combining FTIR and deep learning. By analyzing 371 samples from five provinces, the study found that this method could accurately distinguish the origins of Gastrodia elata, with the ResNet model achieving a recognition accuracy of 100%. To address the issue of spectral distribution shifts caused by environmental and seasonal variations among provinces, spectral standardization, calibration transfer, or meta-learning strategies can be employed to enhance cross-region robustness.

Nevertheless, precise origin tracing of highly similar agricultural products remains challenging, as their subtle chemical and textural differences are easily overlooked by conventional methods. In this context, Cui et al. [57] proposed an innovative approach combining a Twin-Tower Model (TTM) with HSI, employing independent dual-tower architectures to separately extract spectral (Vis–NIR/NIR) and textural features (based on PCA and gray-level co-occurrence matrix), while integrating multi-source information through multi-task learning and optimizing model performance via characteristic wavelength selection. Concurrently, Hu et al. [107] developed a more sophisticated deep learning framework for Ophiopogon japonicus geographical origin identification, utilizing multi-scale 3D convolution (M3DC) to extract spectral-spatial fine-grained features, incorporating spectral-spatial attention mechanisms to dynamically allocate feature weights, and introducing Transformer modules to model long-range dependencies. However, due to domain shifts arising from different growing seasons, environmental conditions, or unseen geographic regions, these methods still exhibit limited generalizability; strategies such as adversarial domain adaptation networks, multi-domain joint training, or few-shot fine-tuning could be employed to enhance cross-region robustness.

#### 4.1.2. Food Adulteration Detection

The issue of sugar adulteration in honey is quite common in food quality testing. Li et al. [128] proposed an effective method to detect sugar contamination in honey by combining mid-infrared (MIR) spectroscopy with CNNs. The study utilized MIR spectral data, with the CNN model automatically extracting features to analyze the differences between pure and adulterated honey and perform classification. The experimental results showed that the CNN model significantly outperformed traditional methods, such as PLS-DA and LS-SVM, in terms of identification performance, achieving an impressive accuracy of 97.96%, especially demonstrating excellent outcomes in market sample applications. Compared to MIR spectroscopy, NMR spectroscopy provides higher-resolution molecular information for detecting honey adulteration. Rachineni et al. [125] proposed an efficient method for detecting sugar adulteration in honey by combining NMR spectroscopy with machine learning. The study analyzed contaminated samples using NMR and applied logistic regression, deep learning, and LGBM classification, obtaining a high accuracy rate.

The fusion of multispectral data can fully leverage the advantages of different spectral data. Chen et al. [156] combined LIBS with Vis–NIR spectroscopy and developed a deep learning network (LVDLNet) to detect Polygonatum and counterfeit products from different geographical sources. This method integrates the elemental information advantage of LIBS with the molecular information advantage of Vis–NIR to significantly improve the identification accuracy. The results showed that the LVDLNet model achieved an accuracy of 98.75% and demonstrated outstanding results in identifying various adulterated samples, surpassing the results of traditional single-model approaches. Regarding the issue of Atlantic salmon adulteration, Li et al. [171] utilized HSI to fuse spectral data from the visible/near-infrared and shortwave infrared bands. The study demonstrated that the combination of CNN with Standard Normal Variate (SNV) preprocessing performed the best, with Vis–NIR spectra outperforming SWIR data in predictive performance.

Nevertheless, the scarcity of adulterated samples is a prevalent phenomenon in food adulteration detection. To address the resulting class-imbalance issue, oversampling, undersampling, or cost-sensitive learning strategies can be employed, alongside generative adversarial networks (GANs) or adversarial domain-adaptation techniques, to enhance robustness against novel adulteration scenarios.

#### 4.1.3. Food Classification Detection

During the slaughter and processing of chicken, blood-related defects (CBDs) in chicken breast are common quality issues. By accurately identifying and classifying these defects, substandard chicken breasts can be promptly detected. Duan et al. [101] combined hyperspectral imaging technology (382.3–1020.2 nm) with CNNs to classify blood-related defects in chicken breasts, including chicken breast congestion (CBC), chicken breast blisters (CBBs), and residual blood in chicken breasts (CBBRs). The study showed that the Faster R-CNN model achieved an average precision (mAP) of 0.990, outperforming the YOLOv4 methods. The identification of wheat varieties is crucial for ensuring their quality and market circulation. Chen et al. [124] combined terahertz time-domain spectroscopy (THz-TDS) technology with CNNs to classify 12 wheat varieties (including hard wheat, medium-strength wheat, and soft wheat). The categorization results showed that the CNN architecture outperformed traditional methods. Addressing food safety detection in mold-contaminated grains, Li et al. [172] implemented short-wave infrared hyperspectral imaging with a hybrid 3D-CNN/2D-CNN architecture to extract spatial-spectral features from maize kernels, obtaining the classification accuracy of 100% in both six-class classification (contamination duration) and binary categorization tasks for aflatoxin contamination. However, the high-dimensional spectral inputs processed by the 3D-CNN can exacerbate overfitting risks—particularly when dataset size or diversity is limited—and should be mitigated via data augmentation or regularization strategies.

For the observation of subtle differences in single-modality spectral data, Zhu et al. [173] focused on identifying slightly sprouted wheat, employing 3D CNN combined with data augmentation techniques to accomplish identification tasks in near-infrared hyperspectral imaging, achieving an accuracy of 98.4%. Given that high-dimensional spectral features can lead to overfitting when sample size is limited, transfer learning and k-fold cross-validation could be employed to further enhance model generalizability. Regarding the classification of fruit varieties, Wang et al. [174] proposed an innovative dual-stream neural network architecture: The local feature branch adopts 1D convolutional neural networks (1D-CNNs) to capture subtle chemical variations between adjacent spectral bands, while the global sequence branch models long-term contextual relationships of spectral bands through bidirectional gated recurrent units (BiGRUs). By leveraging their gating mechanisms to dynamically capture sequential patterns of critical wavelengths, this approach addresses the inherent limitation of conventional CNNs in processing positional information sensitivity. Future research could explore cross-model optimization strategies integrated with spectral fusion technologies to further break through technical bottlenecks in qualitative observation of minute differences.

### 4.2. Quantitative Detection

In the field of intelligent food assessment, quantitative analysis technologies are progressively overcoming the limitations of conventional methods, offering efficient solutions for crop quality evaluation. The qualitative tasks associated with the combination of spectroscopy and deep learning primarily focus on **component** identification, including the detection of heavy metal **concentration**, harmful toxin **levels**, and nutrient **concentration**, among others. Food heavy metal **assessment** is used to assess the levels of harmful metals, such as lead, cadmium, and mercury, in food, ensuring compliance with safety standards. Food harmful toxin content is used to identify and measure the extent of harmful toxin contamination in food, preventing such toxins from compromising food quality and posing a threat to consumer health. Nutrient content analysis focuses on determining the levels of various nutrients (such as proteins, fats, and vitamins) in food, helping to evaluate the nutritional value of the food.

#### 4.2.1. Food Heavy Metal Content Detection

In agricultural production, heavy metal pollution has become a critical issue affecting crop safety and quality. The presence of excessive heavy metals in food poses a severe threat to human health. Long-term consumption of food containing high concentrations of heavy metals can lead to various health problems, including poisoning, kidney damage, and neurological disorders. Therefore, efficient analysis of heavy metal content is essential for reducing food safety risks and ensuring consumer health. Fluorescence hyperspectral technology offers significant advantages in heavy metal detection, enabling rapid, non-destructive, and high-precision analysis, particularly suitable for identifying low-concentration heavy metal contamination. Zhou [13] proposed a method using fluorescence hyperspectral imaging and deep learning to predict lead content in rapeseed leaves. The study employed wavelet transform (WT) and stacked denoising autoencoders (SDAE) to extract deep features from fluorescence spectra, with prediction performed using a support vector regression (SVR) model. Combining the optimal wavelet base function (sym7), this method demonstrated excellent performance in predicting lead content.

Compared to other spectroscopic techniques, LIBS offers unique advantages in heavy metal quantification, and it is capable of directly analyzing solid, liquid, and gas samples for elemental content without the need for complex sample pre-treatment. Lu et al. [175] achieved high-precision recognition of cadmium (Cd) and zinc (Zn) in plants by combining LIBS with explainable deep learning models through their feature-fused convolutional neural network (CARS-CatBoost-CNN) architecture. Furthermore, microwave detection demonstrated unique advantages for liquid food contaminant inspection due to its strong penetration capacity and high moisture sensitivity. CNN models enable direct processing of raw microwave signals without complex preprocessing [176]. The methodology for detecting lead (Pb) contamination in edible oils through microwave phase shift variations essentially aligns with spectral analysis principles, offering novel technical perspectives for spectroscopic applications [177].

#### 4.2.2. Food Harmful Toxin Content Detection

Aflatoxin is a highly carcinogenic substance that is widely present in food, particularly in peanuts, corn, and other crops. Long-term consumption of foods containing aflatoxins can lead to liver damage, immune system suppression, and even increase the risk of cancer. Therefore, timely monitoring and control of aflatoxin levels are crucial for ensuring food safety [178,179]. To effectively detect aflatoxins in peanuts, Zhao et al. [108] proposed a novel pixel-level hyperspectral monitoring technique based on CNN and cumulative learning methods. The study improved the accuracy of aflatoxin measurement by combining different remote sensing datasets and adopted a stepwise learning strategy to overcome issues of data scarcity and low resolution. Experimental results showed that the accuracy of the cumulative learning method exceeded 0.97, significantly outperforming traditional 1D-CNN and transfer learning models, demonstrating its superior performance in aflatoxin analysis in peanuts. Zhu et al. [109] addressed the data imbalance issue by reconstructing the aflatoxin spectra and training the LSTM model, which significantly improved the quantification precision of peanut AFB1. Together, these studies tackle data scarcity via cumulative learning and data imbalance via spectral reconstruction, yet a unified framework to simultaneously address both challenges under varying environmental conditions remains lacking.

Zearalenone (ZEA) is a mycotoxin produced by Fusarium species that widely contaminates cereal crops such as wheat and corn. It is potentially toxic and poses a threat to human and animal health, particularly by causing hormonal disruption and immune system suppression. Therefore, rapid and effective detection of ZEA in wheat is critical for ensuring food security. Nonetheless, key data-related challenges—including limited labeled samples, skewed analyte concentration distributions, and spectral variability due to environmental factors—can impair model generalizability and robustness. To tackle these data-related obstacles, Zhao et al. [164] proposed a method combining a color sensor array with near-infrared spectroscopy and using CNN to detect ZEA in wheat. The study showed that the CNN model performed excellently in quantitative analysis, achieving higher prediction accuracy compared to traditional methods.

Chlorpyrifos is an organophosphorus pesticide commonly used to control pests. Its residue in food crops presents a significant threat to food security. To address the concern of chlorpyrifos contamination in corn oil, Xue and Jiang [16] proposed a determination method by combining Raman spectroscopy with LSTM and CNN models. Their research demonstrated that the LSTM-CNN model performed exceptionally well in predicting chlorpyrifos content, with an R^2^ value of 0.90, though real-world performance still depends on model generalizability across diverse sample conditions.

#### 4.2.3. Food Nutrient Content Detection

Determining the nutrient content of food is essential for ensuring food quality. Taking rice as an example, the anthocyanin content in seeds serves as a critical indicator for assessing nutritional and functional properties. Bao et al. [111] proposed a fusion methodology by integrating deep convolutional generative adversarial networks (DCGAN) with visible-near-infrared hyperspectral imaging, employing a “channel-labeling” strategy to couple spectral data with anthocyanin content for synthetic data generation and accuracy enhancement. Wang et al. [110] primarily focused on identifying anthocyanin content in potatoes using micro-hyperspectral imaging and CNN. The study developed a CNN model based on spectral data, enabling rapid and accurate prediction of anthocyanin content in potatoes.

Similarly, for fruit quality assessment, Kim et al. [82] developed an ensemble neural network-based model for citrus sucrose prediction, which combines stacked species-specific submodules with categorical feature fusion, achieving a 22.1% reduction in root mean square error (RMSE) compared to traditional PLSR methods on multi-variety integrated datasets. Nanda et al. [88] demonstrated a non-destructive detection method for free fatty acids (FFA) in oil palm fruits through the integration of Higuchi fractal dimension and LSTM deep learning with near-infrared spectroscopy. In addition, the SSC of fruits is an important indicator of their sweetness and maturity, which directly affects consumer choices and market pricing. To address the impact of temperature fluctuations on recognition accuracy, Sun et al. [52] proposed a knowledge-guided temperature correction method based on visible/near-infrared spectroscopy for detecting the SSC of watermelon. By combining a 1D-CNN model with temperature-related features using gradient-weighted class activation mapping (Grad-CAM), the method achieved a root mean square error of prediction (RMSEP) 32.5% lower than the global model. This approach provides an effective new way to process temperature interference. The Transformer model, with its self-attention mechanism, can effectively capture long-range dependencies, offering unique advantages for processing the high-dimensional spectral data. Qi et al. [22] combined CNN with Transformer-based hyperspectral imaging technology (900–1700nm) to predict the SSC and pH value of cherry tomatoes. The results showed that the CNN-Transformer model performed excellently in predicting SSC.

Overfitting remains a common issue when applying the above deep learning models to high-dimensional hyperspectral data, especially given the limited number of labeled samples. To address this, several strategies can be adopted. Regularization methods, such as L1/L2 weight penalties and dropout, are widely used to constrain model complexity. Data augmentation techniques, including spectral perturbation, spatial transformations, or random masking, help improve model generalization. In addition, dimensionality reduction methods such as principal component analysis (PCA) or band selection are effective in removing redundant features and reducing input size. These approaches enhance model robustness and are essential for reliable performance in practical hyperspectral applications.

Terahertz spectroscopy has emerged as a promising frontier in analytical technology. Luo et al. [122] investigated terahertz imaging for rice seedling root phenotyping, extracting root length and diameter parameters through image processing and spectral analysis, while validating the technology’s efficiency and accuracy through nitrogen content prediction using linear regression and deep learning models (SSA-SVR achieving optimal performance).

## 5. Conclusions

The synergy of spectral technologies and deep learning demonstrates significant potential for revolutionizing food analysis. While traditional methods, grounded in physicochemical principles, are effective for simple systems, they face limitations with complex mixtures and dynamic variations. Deep learning models (e.g., CNNs, Transformers) overcome these by autonomously extracting hierarchical features from spectral data. Furthermore, multimodal integration (e.g., hyperspectral and Raman) leverages complementary data, achieving superior performance over single-modal approaches.

Despite these advances, critical challenges remain. Fusing heterogeneous spectral data poses difficulties in feature alignment and cross-modal learning. The demand for non-destructive testing and the computational intensity of complex models challenge real-time deployment. Future progress necessitates developing efficient deep learning algorithms that incorporate spectral physics priors for enhanced interpretability, alongside lightweight hardware architectures for practical field use.

Crucially, deep learning-augmented multimodal spectroscopy complements rather than replaces traditional methods. Empirical wavelength selection can inform neural network optimization, while deep learning’s fusion capabilities mitigate errors in complex scenarios inherent to classical approaches. Expanding versatility requires adapting to diverse materials (liquids, porous matrices, tissues) and developing tailored algorithms for resource-limited mobile devices and extreme environments. Flexible multimodal fusion opens new avenues for studying food component dynamics, with architectures like Transformers potentially elucidating constituent interactions.

Collectively, this convergence is driving a paradigm shift from simplistic analysis towards holistic, dynamic systems. Widespread adoption, however, hinges on the balanced co-optimization of data, algorithms, and hardware, coupled with strong interdisciplinary collaboration to bridge the gap between laboratory innovation and industrial scalability.

## Figures and Tables

**Table 1:** Comprehensive comparison of deep learning algorithms.

Method	Type	Key Features	Advantages	Limitations	Typical Applications
DNN	Reg/Cls	FC, nonlinear activations, deep architecture	Learns complex nonlinear mappings, good for structured numerical data	Prone to overfitting, needs large data, high params	Concentration quantification, type identification
CNN	Cls/Reg for Visual Tasks	FC, Convolutional layers, pooling	Efficient for spectral images, captures spatial-spectral features, parameter sharing	Poor for 1D spectra, weak global spectral dependency	Land cover classification, anomaly detection
RNN	Cls/Reg for Sequential Tasks	Recurrent connections, memory cells, temporal dependencies	Captures temporal spectral changes, handles variable-length spectral sequences	Slow training for long sequences, low parallelism	Chemical monitoring, trend prediction
Transformer	Reg/Cls	Self-attention, parallel processing, Enc-Dec Arch	Processes long-range spectral dependencies, parallel training, multi-task adaptability	High memory for high-D spectra, requires massive labeled data	Cross-modal retrieval, spectrum generation
CapsNets	Reg/Cls	CapsVecs encode entities, spatial relations, mag/dir encode continuous values	Encodes spectral spatial hierarchies, robust to spectral shifts, joint multi-task analysis	Complex training for spectra, limited engineering adoption	Protein interaction detection
AE	RL/DR	Enc-Dec Arch, unsupervised latent representation learning	Extracts compact spectral features, suited for unsupervised clustering, spectral anomaly detection	Needs post-processing, reconstruction limits performance	Spectrum dimensionality reduction, spectral denoising
DEC	Clustering	Autoencoder, clustering layer, end-to-end cluster	Improves clustering accuracy, supports regression indirectly, fits high-D data	Requires hyper-tuning, high complexity	Disease spectral subtype identification

Abbreviations: Reg, Regression; Cls, Classification; FC, Fully connected layers; Enc-Dec Arch, encoder-decoder architecture; CapsVecs, Capsule Vectors; mag/dir, magnitude/direction; RL, Representation Learning; DR, Dimensionality Reduction.

**Table 3:** Optical configurations of near-infrared spectroscopy (NIRS).

Mode Name	Principle	Application Scenarios
Diffuse reflectance	Measure scattered light from the sample surface	Commonly used for solids or opaque samples
Transmittance	Measure light passing through the sample	Suitable for semi-transparent or thin samples
Specular reflectance	Capture light reflected at the incident angle	Useful for analyzing smooth surface properties
Directional transmittance	Measure transmitted light in a specific direction	Enhancing quantification of internal microstructure

**Table 4:** Summary table of deep learning models.

Model	Application Domain	Spectral Technique	Performance	Key Advantages
InceptionV3	Potato starch content estimation in tubers	NIR	R^2^ = 0.82, RPD = 2.37	Multi-scale, performance boost, region focus
S-IFCNN	Wolfberry geographical origin discrimination	Vis–NIR–HSI	ACC = 91.99%	Noise robustness, high efficiency
HFA-Net	Quantitative detection of pork freshness	F-HSI, e-nose fusion	R^2^ = 0.9373, RPD = 3.5454	End-to-end fusion, parallel execution
MCRM-CNN	Identification of mould varieties infecting maize kernels	Raman HSI	ACC = 100%	Nonlinear feature extraction, noise suppression
SAE-LSSVM	Prediction of TSS and TA in Kyoho grape	Vis–NIR–HSI	TSS: R^2^ = 0.9237, RPD = 3.25 TA: R^2^ = 0.9216, RPD = 3.21	Size compensation, high generalization
LACNet	Estimating crop LAI and LCC	Vis–NIR–SWIR–HSI	LAI: R^2^ = 0.777 LCC: R^2^ = 0.765	Deep-shallow feature fusion, interpretability
SSAE-CS-SVM	Maize seed variety identification	NIR–HSI	ACC = 95.81%	Noise robustness, online detection potential
FP-YOLOv5	Early bruise detection on apples	SWIR–HSI	mAP = 98.2%	Real-time detection, enhanced contrast, lightweight model
CNN-Transformer	SSC and pH prediction of cherry tomatoes	NIR–HSI	SSC: R^2^ = 0.83 pH: R^2^ = 0.60	Interpretability, noise robustness
CLNet	Prediction of protein and moisture content in sorghum grains	NIR–SWIR–HSI	Protein: R^2^ = 0.987, RPD = 7.1949 Moisture: R^2^ = 0.9983, RPD = 24.3681	High robustness, real-time potential
CNN-LSTM	Monitoring of gel strength, WHC, and whiteness in surimi	Vis–NIR–HSI	Gel strength & whiteness: R^2^ > 0.92, WHC: R^2^ > 0.55	Multi-indicator prediction, noise robustness
CNN-LSTM	Discrimination of sulfur-fumigated lilies & prediction of nutrient contents	Vis–NIR–SWIR–HSI	Sulfur fumigation discrimination ACC = 97.3% Polysaccharides: R^2^ = 0.769, total phenols: R^2^ = 0.699, SO_2_: R^2^ = 0.755	High-dimensional data handling, noise robustness, potential for real-time deployment
Tran-MPRNet, CNN	Geographical origin identification of beef	Vis–NIR–HSI	Tran-MPRNet: R^2^ = 0.973, CNN: ACC = 91.01%, mobile app validation: ACC = 91.67%	Real-time mobile deployment, small-data robustness

**Abbreviations:** RPD, Ratio of Performance to Deviation; ACC, testing accuracy; TSS, total soluble solids; TA, titratable acidity; LAI, leaf area index; LCC, leaf chlorophyll content; mAP, mean Average Precision; SSC, Soluble solid content; WHC, water-holding capacity.

## Data Availability

No new data were created or analyzed in this study. Data sharing is not applicable to this article.
